# Assessing the performance of artificial intelligence models in evaluating inflammatory skin disease severity: a systematic review and meta-analysis

**DOI:** 10.1093/bjd/ljaf250

**Published:** 2025-06-26

**Authors:** Zhuo Ran Cai, Jiyeong Kim, Shawheen J Rezaei, Michael L Chen, Fadi Touma, Catherine Zhu, Sonia Onyeka, Fonette Fonjungo, Jesutofunmi A Omiye, Isabelle Krakowski, Lotanna Nwandu, Robert Biossonnette, Justin M Ko, Eleni Linos

**Affiliations:** Department of Dermatology, School of Medicine, Stanford University, Stanford, CA, USA; Center for Digital Health, School of Medicine, Stanford University, Stanford, CA, USA; Department of Dermatology, School of Medicine, University of Montreal, Montreal, QC, Canada; Department of Dermatology, School of Medicine, Stanford University, Stanford, CA, USA; Center for Digital Health, School of Medicine, Stanford University, Stanford, CA, USA; Department of Dermatology, School of Medicine, Stanford University, Stanford, CA, USA; Center for Digital Health, School of Medicine, Stanford University, Stanford, CA, USA; Department of Dermatology, School of Medicine, Stanford University, Stanford, CA, USA; Center for Digital Health, School of Medicine, Stanford University, Stanford, CA, USA; Faculty of Medicine and Health Sciences, McGill University, Montreal, QC, Canada; Faculty of Medicine and Health Sciences, McGill University, Montreal, QC, Canada; Department of Dermatology, School of Medicine, Stanford University, Stanford, CA, USA; Center for Digital Health, School of Medicine, Stanford University, Stanford, CA, USA; Meharry Medical College, Nashville, TN, USA; Department of Dermatology, School of Medicine, Stanford University, Stanford, CA, USA; Center for Digital Health, School of Medicine, Stanford University, Stanford, CA, USA; Department of Biomedical Data Science, School of Medicine, Stanford University, Stanford, CA, USA; Department of Dermatology, School of Medicine, Stanford University, Stanford, CA, USA; Center for Digital Health, School of Medicine, Stanford University, Stanford, CA, USA; Department of Oncology and Pathology, Karolinska Institutet, Stockholm, Sweden; Carle Illinois College of Medicine, Champaign-Urbana, IL, USA; Innovaderm Research, Montreal, QC, Canada; Department of Dermatology, School of Medicine, Stanford University, Stanford, CA, USA; Department of Dermatology, School of Medicine, Stanford University, Stanford, CA, USA; Center for Digital Health, School of Medicine, Stanford University, Stanford, CA, USA

## Abstract

**Background:**

Artificial intelligence (AI) applications in dermatology have expanded beyond diagnosis and have shifted towards assessing disease severity.

**Objectives:**

To qualitatively and quantitatively evaluate the performance of image-based AI models in severity assessment for various skin diseases.

**Methods:**

In this systematic review and meta-analysis, we collected studies using four electronic databases, including PubMed, Embase, Institute of Electrical and Electronics Engineers Xplore and Web of Science, published from 1 January 2017 to 6 April 2023, and updated the search in November 2023. Studies assessing the performance of deep learning AI models on the severity of skin diseases were included. We excluded studies that utilized a nonvalidated severity index, lacked clinical images, and assessed wounds, ulcers or burns. Two independent reviewers extracted prespecified study characteristics for the summary table. For the meta-analysis, contingency tables were extracted, when possible, and reconstructed for each severity measure. Accuracy was calculated using a bivariate model in Metandi package, and meta-regression was performed by disease type and scoring system. This study was registered with PROSPERO (CRD42023487228).

**Results:**

Our initial search identified 7737 records. After duplicate removal and abstract screening, we reviewed the full text of 192 articles and included 45 studies for systematic review and 19 for meta-analysis. The pooled sensitivity and specificity of AI models were 80.5% [95% confidence interval (CI) 76.2–84.2] and 96.2% (95% CI 94.9–97.2), respectively. Moreover, pooled sensitivity differed by disease (atopic dermatitis 91.8% vs. acne 80.7%, *P* = 0.005; acne 80.4% vs. psoriasis 71.1%, *P* = 0.044) and scoring system [Eczema Area and Severity Index 97.3% vs. Investigator’s Global Assessment (IGA) for atopic dermatitis 78.9%, *P* < 0.001; Hayashi Grading 89.7% vs. IGA for acne 69.8%, *P* < 0.001].

**Conclusions:**

Our findings show that current AI models exhibit a high level of capacity in disease severity assessment. Nevertheless, efforts are urgently needed to improve transparency in data reporting and conduct high-quality prospective studies using objective reference standards in clinical settings to generate reliable evidence.

## Teaser

This systematic review and meta-analysis evaluated the performance of image-based artificial intelligence (AI) models in assessing the severity of skin diseases by analysing data from four electronic databases covering studies published since January 2017. The pooled sensitivity and specificity of the AI models were found to be 80.5% and 96.2%, respectively, with performance varying by disease type and scoring system. Although the findings demonstrate the high capability of AI models in severity assessment, there is a need for improved transparency in data reporting and higher-quality prospective studies using objective reference standards in clinical settings.

What is already known about this topic?

- Previous systematic reviews have been performed on the use of technological innovations in skin disease severity assessment.
- One review assessed machine learning applications for psoriasis evaluations using skin images, including 12 studies, of which more than half were published between 1998 and 2016.
- Another study reviewed the performance of technology (e.g. actigraphy) to evaluate scratching behaviour in patients with pruritus.
- The most recent review qualitatively synthesized studies that examined artificial intelligence (AI) models for different skin disease detection.

What does this study add?

- None of these previous three review articles included meta-analyses. We performed a meta-analysis to quantify the overall accuracy and reliability of AI models for severity assessments.
- We also found that the pooled sensitivity and specificity differed by disease type, scoring system and severity level.
- These novel findings provide quantitative insights into the trajectories of AI models in development and research for practical use in severity assessment.

There is increased interest in the medical application of artificial intelligence (AI) technologies, including within dermatology. Skin diseases are a leading contributor to the global burden of disease and affect more than 20% of the world’s population.^1,2^ AI can serve as a powerful tool to improve dermatological care. To date, most research on image-based AI models in dermatology has focused on the classification of malignancies, particularly in diagnosing skin cancer.^3^ These studies have leveraged AI to improve detection and expand access to care. Research has also explored AI applications in diagnosing nonmalignant skin diseases, including inflammatory conditions. Some models have been developed to differentiate between common inflammatory dermatoses and provide differential diagnoses.^4,5^

Recently, AI applications within dermatology have expanded to grading the severity of inflammatory diseases such as psoriasis and atopic dermatitis.^6^ Severity-based patient care improves treatment outcomes and patient satisfaction and reduces healthcare costs.^7^ Severity scoring systems are routinely used to assist clinicians with treatment decisions and to evaluate treatment efficacy in practice.^8^ However, the high interrater discrepancy between clinicians in classifying severity has been a longstanding challenge in dermatology. AI tools are poised to augment clinicians’ capabilities in grading the severity of skin disease with the potential of improving precision, which would contribute to improving disease outcomes. Despite their importance, the synthesized evidence for the performance of AI models in skin disease severity assessment has been underinvestigated.

Prior systematic reviews^9–11^ and meta-analyses^12,13^ have concentrated on the detection of skin cancer using image-based AI models, with a recent review including 64 studies, of which 52 were focused on detection and 12 concentrated on severity assessment.^14^ Notably, a meta-analysis evaluating the performance of image-based AI models for skin disease severity assessment has not been reported.^15,16^ To address this gap, our study aims to quantitatively assess the effectiveness of AI models in classifying the severity of skin diseases through a meta-analysis, and critically appraise the characteristics of these AI models through a systematic review.

## Materials and methods

### Search strategy and selection criteria

In this systematic review and meta-analysis, we searched for peer-reviewed articles on image-based AI algorithms assessing disease severity in inflammatory skin disease from 1 January 2017 to 6 April 2023 (updated search on 10 November 2023). We explored databases including PubMed, Embase, Institute of Electrical and Electronics Engineers Xplore and Web of Science, focusing on deep learning AI algorithms, skin diseases and disease severity (Appendix S1; see Supporting Information). Specifically, we targeted AI models using convolutional neural networks. We included studies published since 2017 because of the significant advancements in AI algorithm performance.^4^ Conference proceedings from the Annual Conference on Neural Information Processing Systems, the Hawaii International Conference on System Sciences, the International Conference on Machine Learning, the International Conference on Learning Representations, the Association for the Advancement of Artificial Intelligence, the Conference on Computer Vision and Pattern Recognition, the Annual Conference on Health, Inference, and Learning and Machine Learning for Health were manually searched. Google Translate was used for screening articles that were not written in the English language. If essential data were not reported, we contacted the authors, asking them to provide this information.

We structured the eligibility criteria using the population, index test, reference standard and disease of interest framework. We included studies on all ages, sexes and races/ethnicities. The disease of interest was skin, hair or nail diseases of any severity across skin types, and we included studies that used AI algorithms to assess disease severity based on clinical images with any types of outcome measures (e.g. accuracy, sensitivity and specificity). We excluded studies that evaluated disease severity using a nonvalidated index, in addition to those focused solely on the accuracy of image segmentation. Studies focusing on skin tumours, wounds, burns or photoageing were excluded. Studies that employed any index tests were included. We did not limit the type of reference standards. Reviews, editorials and case reports were excluded.

Eight reviewers (Z.R.C., J.K., F.T., S.J.R., F.F., S.O., J.A.O., I.K.) ascertained eligibility assessment, extracted data and evaluated study quality. Two reviewers independently screened titles, abstracts and full texts and extracted prespecifed data. Disagreements were resolved by a third reviewer. We used Covidence (Melbourne, VIC, Australia) for duplicate removal, screening, and full-text review, and Excel (Microsoft, Redmond, WA, USA) for data extraction. Z.R.C. entered data into the summary table, which were verified by J.K. and S.J.R. Data were prepared by J.K. for meta-analysis, which were double-checked by Z.R.C. and M.L.C. All co-authors reviewed the final results. The study followed the PRISMA diagnostic test accuracy guidelines (Appendix S2; see Supporting Information) and was registered with PROSPERO (CRD42023487228).

### Data extraction

We extracted study characteristics, including target condition, severity index, outcome measures, study design, data source, number of images (training, validating and testing), reference standard, AI algorithms and validation. Additionally, we assessed skin tone diversity based on Fitzpatrick skin type and race/ethnicity. Primary outcome measures were accuracy, sensitivity and specificity, with accuracy being the most common, followed by intraclass correlation coefficient (ICC). When available, we extracted contingency tables (true positive, false positive, false negative and true negative) to compute pooled sensitivity and specificity for meta-analysis. A positive result matched the AI’s severity classification with the reference standard, whereas a negative result indicated a discordance. We generated multiple contingency tables for each severity level, assuming independence to evaluate AI performance across different skin conditions rather than derive a precise accuracy measure for a single disease.

### Data synthesis

We calculated pooled sensitivity and specificity with 95% confidence intervals (CIs) of AI algorithms in disease severity assessment using the bivariate model of the Metandi package in Stata 18 (College Station, TX, USA).^17,18^ We plotted summary receiver operating characteristic (SROC) curves to visualize sensitivity and specificity with 95% confidence and prediction regions. To accommodate study variability from different diseases, severity scores and AI models, we opted for a random effects model. For heterogeneity, we visually inspected and examined the SROC. Subgroup analyses examined accuracy variations by severity level. Atopic dermatitis severity was reported using the Eczema Area and Severity Index (EASI) and Investigator’s Global Assessment (IGA), combined into grades 0–3. Acne severity was reported using IGA (grades 0–4), Hayashi Grading, and New Acne Severity (mild, moderate, severe and very severe), regrouped into three levels [IGA grades 0–1 (mild), IGA grade 2 (moderate), IGA grades 3–4 (severe/very severe)]. This provides an overview of the accuracy of AI algorithms by acne severity, when possible, although it does not generate a precise point estimate per severity level. Moreover, we performed bivariate meta-regression to compare AI model accuracy by disease type (atopic dermatitis, acne and psoriasis) and scoring system (EASI, IGA, Hayashi Grading/New Acne Severity) using the Meqrlogit package in Stata 18.^19^ As a secondary outcome, we evaluated AI reliability in assessing disease severity by calculating pooled ICCs for studies that reported it.

### Quality assessment

We assessed the risk of bias in each study using QUADAS-2, focusing on patient selection, index test, reference standard, and flow and timing. Two reviewers conducted assessments independently, resolving conflicts with a third reviewer (Z.R.C., J.K., M.L.C. and L.N.). We calculated κ statistics for interrater agreement and computed the Spearman correlation coefficient between sensitivity and specificity to check for a positive threshold effect.^20^ Sensitivity analyses included excluding potential outliers and studies rated as unclear or high-bias in any domain.^21^ Publication bias was evaluated using Deeks’ Funnel Plot Asymmetry Test, regressing funnel plots against effective sample size and diagnostic odds ratio.^22^

## Results

**Figure 1:**
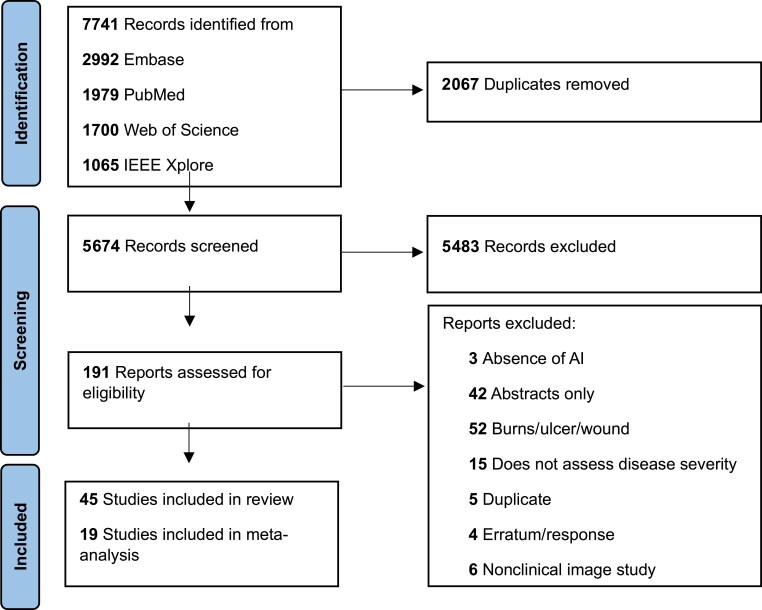
Study selection. AI, artificial intelligence; IEE, Institute of Electrical and Electronics Engineers.

Initially, 7741 records were retrieved, and we screened 5674 after excluding duplicates (Figure 1). We further excluded 5483 studies and reviewed 191 full texts. We included 45 studies in the systematic review^6,23–66^ and 19 studies in the meta-analysis for accuracy (12 studies with 120 contingency tables)^26, 28, 30, 36, 39, 53, 56, 61–63^ and reliability (8 studies with 24 ICCs).^23,24,29,37,41,45,56,65^ One study provided both accuracy and reliability data.^56^ Studies from previous reviews were included if eligible (*n* = 13).^26,29,31–33,39,41,43,45,56,57,63,65^ Studies included for meta-analysis are provided in Appendix S3 (see Supporting Information).

Appendix S4 (see Supporting Information) shows the study characteristics of the included studies. Most studies were conducted in Asia (64%, 29 of 45), followed by Europe (29%, 13 of 45), Australia (4%, 2 of 45) and North America (2%, 1 of 45). Eleven inflammatory skin diseases met our inclusion criteria with the most frequent conditions being psoriasis (31%, *n* = 14 of 45), acne (27%, 12 of 45) and atopic dermatitis (9%, 4 of 45). Among 23 severity scales, Psoriasis Area Severity Index (PASI) was the most common (31%, 14 of 45), followed by Hayashi Grading (11%, 5 of 45) and IGA for acne (9%, 4 of 45).

Most studies provided information on model architectures (91%, 41 of 45) and used private datasets (73%, 33 of 45) to train and test their algorithms. A detailed breakdown of training and testing sets was mostly available (93%, 42 of 45). Demographic information (31%, 14 of 45) and skin tone metadata (22%, 10 of 45) were not commonly reported. Over half of the studies (51%, 23 of 45) included class distribution data, referring to the balance of different severity levels in their datasets. Class distribution is important in AI because imbalanced datasets, where one severity level is overrepresented, can bias model performance and limit generalizability. Expert annotations were the reference standard for nearly all studies (98%, 44 of 45), except for one that used histopathology diagnosis. Only one study applied a prospective design, while the rest were performed in an experimental nonclinical setting or proof-of-concept settings using retrospective data. Most studies (69%, 31 of 45) validated their models using an independent test set from the same source as the training set, 4 used test sets from different sources, and 10 had incomplete validation processes (Appendices S5, S6; see Supporting Information). In our methodological quality assessments, one-third of the studies showed a high risk of bias (*n* = 14), although the applicability concern was low in most studies (*n* = 43). Overall QUADAS-2 agreement rate was low (κ = 0.27) and major disagreement originated from the risk of bias assessment (Appendix S7; see Supporting Information). All the related reviewers agreed with the final assessments.

**Figure 2:**
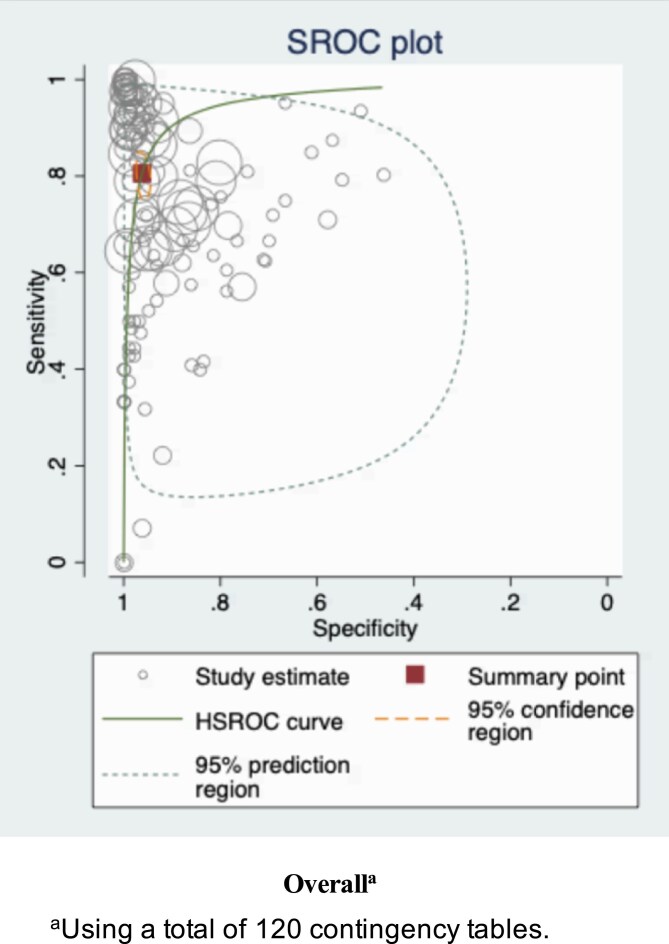
Summary receiver operating characteristic (SROC) plot (overall). HSROC, hierarchical SROC.

The pooled sensitivity and specificity of the image-based AI algorithms were 80.5% (95% CI 76.2–84.2) and 96.2% (95% CI 94.9–97.2), respectively. The summary estimates were plotted on the SROC curve, where the narrow confidence region is observed while the prediction region is wide, indicating large heterogeneity (Figure 2).

**Figure 3:**
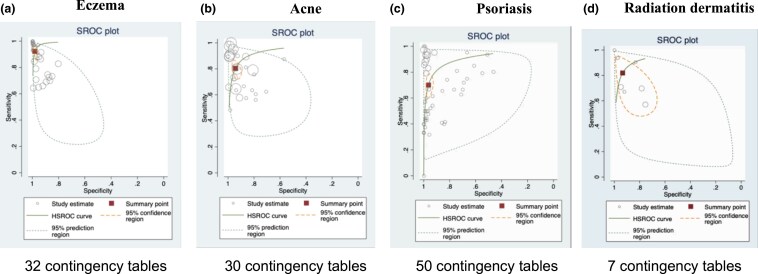
Summary receiver operating characteristic (SROC) plots by disease. (a) Eczema (32 contingency tables). (b) Acne (30 contingency tables). (c) Psoriasis (50 contingency tables). (d) Radiation dermatitis (7 contingency tables). HSROC, hierarchical SROC.

The meta-regression showed that the sensitivity of AI algorithms in severity assessment significantly differed by disease type and scoring system (Table 1). Pooled sensitivity was highest in atopic dermatitis, followed by acne, and lowest in psoriasis (atopic dermatitis vs. acne: 91.8% vs. 80.7%, *P* = 0.005; acne vs. psoriasis: 80.4% vs. 71.1%, *P* = 0.044). Specificity was also highest in atopic dermatitis (atopic dermatitis vs. acne: 98.1% vs. 94.3%, *P* = 0.014). (Figure 3). When we assessed the meta-regression results by atopic dermatitis scoring system, EASI showed higher accuracy than IGA (sensitivity 97.3% vs. 78.9%, *P* < 0.001; specificity 99.3% vs. 94.4%, *P* < 0.001), respectively. For acne, Hayashi Grading/New Acne Severity had higher accuracy than IGA both in sensitivity (89.7% vs. 69.8%, *P* < 0.001) and specificity (97.4% vs. 89.6%, *P* = 0.003). Through the subgroup analyses, the accuracy also differed by the severity level (Table 2). For acne, specificity was higher for the most severe grade (94.9%, 95% CI 91.5–97.0) than for the moderate grade (82.0%, 95% CI 71.1–89.4). There was no evidence of a small-study effect for accuracy in the regression test asymmetry (*P* = 0.96). Deeks’ funnel plot can be found in Appendix S8 (see Supporting Information). A threshold effect appeared to be present for psoriasis (Spearman’s ρ = −0.28, *P* = 0.049), which could account for the high heterogeneity of our findings along with accuracy difference by disease type. In sensitivity analyses, the pooled sensitivity and specificity barely changed (< 1%) when outliers or low-quality studies were excluded, yet sensitivity increased to 85.4% when we excluded both outliers and low-quality studies (Appendix S9; see Supporting Information). The pooled reliability was 78.0% (95% CI 72.2–83.8) in disease severity assessment (Appendix S10; see Supporting Information).

**Table 1:** Meta-regression results by disease types and scoring systems

	Disease type		*P*-value
	Atopic dermatitis (*N* = 32)	Acne (*N* = 30)	
SE	91.8 (87.8–94.5)	80.7 (72.9–86.7)	0.005
SP	98.1 (96.8–98.9)	94.3 (90.7–96.5)	0.014
	Atopic dermatitis (*N* = 32)	Psoriasis (*N* = 50)	
SE	91.8 (87.7–94.6)	71.7 (63.3–78.8)	< 0.001
SP	98.1 (96.5–99.0)	96.0 (93.5–97.6)	0.07
	Acne (*N* = 30)	Psoriasis (*N* = 50)	
SE	80.4 (73.6–85.8)	71.1 (63.9–77.3)	0.044
SP	94.6 (90.4–97.0)	96.1 (93.7–97.6)	0.421
	Scoring system		
Atopic dermatitis	EASI (*N* = 16)	IGA^a^ (*N* = 16)	
SE	97.3 (95.7–98.3)	78.9 (72.3–84.3)	< 0.001
SP	99.3 (98.7–99.7)	94.4 (91.0–96.6)	< 0.001
Acne	Hayashi Grading and New Acne Severity (*N* = 13)	IGA^b^ (*N* = 17)	
SE	89.7 (85.1–92.9)	69.8 (62.2–76.4)	< 0.001
SP	97.4 (95.3–98.6)	89.6 (83.6–93.6)	0.003
Radiation dermatitis^c^	CTCAE^d^ (*N* = 3)	RTOG^e^ (*N* = 4)	
SE	66.3 (48.9–80.2)	88.5 (75.2–95.1)	0.020
SP	85.6 (60.5–95.9)	96.4 (87.6–99.0)	0.225

CTCAE, Common Terminology Criteria for Adverse Events; EASI, Eczema Area and Severity Index; IGA, Investigator’s Global Assessment; *N*, number of contingency tables; RTOG, Radiation Therapy Oncology Group; SE, sensitivity; SP, specificity. ^a^IGA for atopic dermatitis; ^b^IGA for acne vulgaris; ^c^sample size of radiation dermatitis was small. Hence, the interpretation of this result may need caution. Pooled sensitivity was 81.9% [95% confidence interval (CI) 64.4–91.9] and pooled specificity was 93.5% (95% CI 82.0–97.8). ^d^CTCAE grading for skin toxicity; ^e^RTOG grading criteria for skin toxicity. Data are presented as % (95% CI) unless otherwise stated.

**Table 2:** Subgroup analysis results by disease types and severity levels

Severity levels				
Atopic dermatitis^a^	EASI 0/IGA 0 (*N* = 8)	EASI 1/IGA 1 (*N* = 8)	EASI 2/IGA 2 (*N* = 8)	EASI 3/IGA 3 (*N* = 8)
SE	96.6 (90.3–98.8)	86.8 (73.7–93.9)	89.8 (77.4–95.7)	92.8 (80.2–97.6)
SP	99.2 (97.3–99.7)	96.8 (92.2–98.7)	94.6 (87.7–97.7)	99.1 (97.4–99.7)
				
Acne^b^	Severity I (*N* = 8)	Severity II (*N* = 7)	Severity III (*N* = 10)	
SE	74.5 (64.6–82.3)	75.9 (66.0–83.5)	73.4 (62.1–82.2)	N/A
SP	94.4 (86.3–97.8)	82.0 (71.1–89.4)	94.9 (91.5–97.0)	N/A
Psoriasis^c^	PASI Grade 0 (*N* = 11)	PASI Grade 1 (*N* = 11)	PASI Grade 2 (*N* = 11)	PASI Grade 3 (*N* = 11)
SE	60.6 (38.8–78.9)	78.9 (66.3–87.7)	73.4 (62.2–82.2)	60.3 (46.0–73.1)
SP	98.5 (97.1–99.3)	82.2 (69.6–90.2)	85.9 (73.4–93.0)	98.0 (96.0-99.0)

EASI, Eczema Area and Severity Index; IGA, Investigator’s Global Assessment; *N*, number of studies; PASI, Psoriasis Area and Severity Index; SE, sensitivity; SP, specificity. ^a^EASI and IGA for atopic dermatitis. ^b^For acne, three scoring systems were combined (IGA for acne vulgaris, New Acne Severity and Hayashi Grading). Severity I includes grades 0–1 from IGA and mild from New Acne Severity and Hayashi Grading. Severity II includes grade 2 from IGA and moderate from New Acne Severity and Hayashi Grading. Severity III includes grades 3–4 from IGA and severe and very severe from New Acne Severity and Hayashi Grading. In the acne subgroup analysis, five acne measurements used a Combined Global Criterion owing to the difficulty to be properly combined with other measurements. ^c^For psoriasis, the PASI score was used to evaluate disease severity. Erythema, induration and desquamation are each assessed as none (Grade 0), mild (Grade 1), moderate (Grade 2), severe (Grade 3) or very severe (Grade 4) for different body areas. In the psoriasis subgroup analysis, the very severe grade was excluded because of a small sample size (*n* = 6). Data are presented as % (95% CI) unless otherwise stated.

## Discussion

In this systematic review of 45 studies, including a meta-analysis of 19 studies, we found that image-based AI algorithms accurately and reliably assess the severity of inflammatory skin diseases. Our findings show a high pooled specificity of 96.2% for severity assessment, surpassing the 93.9% specificity reported for disease detection from medical imaging in a prior meta-analysis.^67^ This underscores the strong performance of AI, not just in identifying diseases, but also in evaluating their severity.

Our findings demonstrate the promising capacity of current AI models to closely mimic the performance of dermatologists in severity assessment. However, accurately defining disease severity remains challenging owing to the inherent variability in clinician-based evaluations and the limitations of existing scoring systems, which often fail to fully capture patient experiences. Clinical trials commonly utilize objective physician scoring systems such as PASI, EASI and IGA, alongside patient-reported outcome measures (PROMs) such as the Dermatology Life Quality Index and Peak Pruritus Numerical Rating Scale (PP-NRS). Notably, none of the studies included in our review directly compared AI-based severity assessments with PROMs. Given these limitations, we recommend that future research incorporates PROMs in the development and validation of AI models.

Different accuracy levels by disease type and scoring system indicate that it is somewhat easier to replicate dermatologists’ performance for some disease types (atopic dermatitis and acne) and scoring systems (EASI and Hayashi Grading/New Acne Severity) than others. It is encouraging to see the promising performance of AI models in classifying the most severe and least severe grades in psoriasis. One possible implication from our findings is that less experienced medical providers in dermatology could benefit from this AI assistance in severity assessment. AI tools can educate medical students and residents by highlighting key clinical features and providing real-time feedback. Such AI models can also aid timely treatment decisions, especially for physicians without dermatologist access. AI offers objective severity assessments, identifying cases that need aggressive intervention, ensuring optimal care in resource-limited settings. For experienced clinicians, AI ensures consistent severity assessments across providers. However, further effort is warranted to improve the performance of classifying the middle grades, which are more challenging and, hence, could be more useful. Overall, this study contributes novel quantitative insights about future directions of AI algorithms in research developments in addition to the potential benefits of AI models in triaging patient care by disease severity. We call for further studies that explore the validation of these algorithms on a more diverse population and examine the testing of these algorithms on prospective datasets.

In our systematic review, accuracy was the primary metric, with acne, psoriasis and atopic dermatitis as the most studied diseases for severity assessment, which was consistent with previous findings.^14^ An earlier review of deep learning image analyses by Choy *et al*. noted the highest accuracy in psoriasis (93–100%, *n* = 2) and the lowest in acne (67–86%, *n* = 4), but lacked a meta-analysis for direct comparisons.^14^ Most studies in our review used private datasets with little metadata reporting. Important information such as Fitzpatrick phototype, demographic information or the number of images per patient, was often missing, which made it challenging to assess dataset diversity. AI model reliability depends on training data; models trained mostly on light skin tones often underperform on darker tones. Additionally, AI model performance may be adversely affected by limited images per patient, which reduces generalizability and robustness. Models developed from small or insufficiently diverse datasets are more prone to overfitting and might fail to fully capture disease variability, thus limiting their real-world applicability. Most studies were conducted in Asia and Europe, indicating a need for more demographic diversity in training data.^68^ Previous reviews of image-based AI algorithms have consistently raised concerns about the lack of transparency in metadata reporting.^69^ Efforts to standardize data reporting include the TRIPOD-AI guidelines. New quality assessment and reporting frameworks, such as PROBAST-AI, PRISMA-AI and QUADAS-AI, are being developed to enhance the quality, reproducibility and transparency of AI research in medicine.^69,70^ Additionally, few studies used prospective designs. While we assessed the promising performance of image-based AI algorithms mainly through retrospective studies, we recommend that future research employs prospective or randomized designs in real clinical settings, which helps to reduce bias associated with curated datasets, better reflects real-world performance and provides insights into how AI can be integrated into clinical workflows. Future studies should also explore AI severity assessment using full-body images for more generalizable evidence.

The strengths of our study include multiple subgroup analyses and meta-regressions that generated novel knowledge about the variability of accuracy by disease type, scoring system and severity level, which has important implications for clinical practice and research. In addition, we performed two types of meta-analyses in this study; one for accuracy using contingency tables and another for reliability using ICCs. We observed consistent performance of AI algorithms when we compared the pooled sensitivity (80%) and pooled reliability (78%). Moreover, the sensitivity analyses revealed that the findings were rigorous and mostly stayed the same when outliers and low-quality studies were removed. Lastly, we were able to identify potential sources of heterogeneity in our accuracy meta-analysis, including disease type, scoring systems or threshold effect in psoriasis.

Our study has some limitations. Firstly, owing to varying outcome measures, our analysis was limited to studies providing contingency tables for accuracy or ICCs as reliability, resulting in pooled metrics from a subset of studies. Some studies provided multiple contingency tables, and we treated such tables independently. Thus, these metrics should be interpreted as overall estimates rather than precise ones. Secondly, for acne severity, we combined different scoring systems for subgroup analysis, which might not accurately represent model performance. Thirdly, high heterogeneity suggests caution in generalizing our accuracy findings. Despite no small-study effect or publication bias impacting accuracy, a small-study effect in reliability assessments indicates potential variability, although correcting for this bias probably improved reliability. Fourthly, more than 75% of studies (78%, 35 of 45) did not report skin tone data, preventing us from assessing how well AI models perform across diverse populations. Without better skin tone representation, AI severity assessment tools risk worsening health disparities, especially for darker skin tones.^71^ For example, erythema, a common marker of inflammation, is frequently underestimated and may appear more violaceous and grey in individuals with darker skin tone.^72,73^ Therefore, exploring the use of objective reference standards such as colorimetry could be valuable for enhancing AI performance in disease severity assessments. Employing objective measures may enable assessments of absolute accuracy rather than relying solely on relative accuracy, which only reflects how closely models align with clinician evaluations. Future research must prioritize reporting skin tone in AI model development. High-quality, diverse dermatology datasets, including individuals with darker skin tones, are urgently needed through international collaborations. Without these efforts, the clinical utility of AI models will remain uncertain for many individuals worldwide.

Our findings from a systematic review and meta-analysis show that image-based AI algorithms have substantial potential in assessing the severity of various inflammatory skin diseases, which affect more than 20% of the world’s population.^1^ While this study provides evidence-based insight into the applications of AI algorithms for severity-based patient care, we also acknowledge the data quality limitations from studies with poor design, inadequate reporting or possible bias. This study highlights the urgent need to improve transparency in data reporting and conduct high-quality prospective studies using an objective reference standard in clinical settings to generate better-quality evidence. Taken together, these endeavours will support the development and implementation of reliable and clinically useful AI tools that are ultimately projected to enhance clinicians’ performance and improve patient outcomes.

## Supplementary Material

ljaf250_Supplementary_Data

## Data Availability

The datasets used and/or analysed during the current study are available from the corresponding author on reasonable request.
