# Supplementary material for: Assessing the performance of artificial intelligence models in evaluating inflammatory skin disease severity: a systematic review and meta-analysis
Source: Br J Dermatol. 2025 Jun 26;193(5):847–55. doi: 10.1093/bjd/ljaf250 (PMC12532143; doi:10.1093/bjd/ljaf250)
Supplement: ljaf250_Supplementary_Data [file ljaf250_supplementary_data.docx]

**Supplementary materials**

Appendix 1. Full search terms

Appendix 2. PRISMA-DTA

Appendix 3. Studies included for meta-analysis

Appendix 4. Summary of characteristics of included studies

Appendix 5. Additional information on study characteristics

Appendix 6. Number of images, capturing modalities and photography types of included studies

Appendix 7. Risk of bias assessment (QUADAS-2)

Appendix 8. Deek’s Regression Asymmetry Funnel Plot for accuracy

Appendix 9. Sensitivity analyses for accuracy

Appendix 10. Results for reliability: Asymmetry funnel plot and sensitivity analyses for reliability

Appendix 11. Studies excluded in the full-text review stage

**Appendix 1. Full search terms for PubMed and Embase**

**PubMed Search**

| Set # | Concept | Syntax |
| --- | --- | --- |
| 1 | Artificial Intelligence, machine learning, neural networks | "Artificial Intelligence" [mesh] OR "Neural Networks, Computer"[Mesh] OR "Artificial Intelligence" [tw] OR "Neural Network" [tw] OR "Neural Networks"[tw] OR "Deep Learning" [tw] OR "Machine Learning" [tw] OR “algorithm*”[tw] OR “augmented intelligence”[tw] |
| 2 | Dermatology and skin diseases | "Skin Diseases"[Mesh] OR "Dermatology"[Mesh] OR derm*[tw] OR skin[tw] OR psoriasis[tw] OR alopecia[tw] OR acne[tw] OR cutaneous[tw] |
| 3 | Disease severity | "Patient Acuity"[Mesh] OR "Severity of Illness Index"[Mesh] OR severity[tw] OR severities[tw] OR assessment[tw] OR “patient acuity”[tw] OR measurement[tw] |
| 4 | combining | #1 AND #2 AND #3 |
| 5 | Limits- 2017 to present, no literature reviews or case reports | #4 AND 2017:2023[dp] NOT ("Case Reports" [Publication Type] OR "Review" [Publication Type] OR "Editorial" [Publication Type]) |

**Embase Search**

| Set # | Concept | Syntax |
| --- | --- | --- |
| 1 | Artificial Intelligence, machine learning, neural networks | 'artificial intelligence'/exp OR 'machine learning'/exp OR 'Artificial Intelligence':ti,ab,kw OR 'Neural Network':ti,ab,kw OR 'Neural Networks':ti,ab,kw OR 'Deep Learning':ti,ab,kw OR 'Machine Learning':ti,ab,kw OR 'algorithm*':ti,ab,kw OR 'augmented intelligence':ti,ab,kw |
| 2 | Dermatology and skin diseases | 'skin disease'/exp OR 'dermatology'/exp OR derm*:ti,ab,kw OR skin:ti,ab,kw OR psoriasis:ti,ab,kw OR alopecia:ti,ab,kw OR acne:ti,ab,kw OR cutaneous:ti,ab,kw |
| 3 | Disease severity | 'severity of illness index'/exp OR 'patient acuity'/exp OR 'Eczema Area and Severity Index'/exp OR 'Nottingham Eczema Severity Score'/exp OR 'Psoriasis Area and Severity Index'/exp OR 'Psoriasis Severity Index'/exp OR 'skin disease assessment'/exp OR 'Severity Scoring of Atopic Dermatitis'/exp OR severity:ti,ab,kw OR severities:ti,ab,kw OR assessment:ti,ab,kw OR 'patient acuity':ti,ab,kw OR measurement:ti,ab,kw |
| 4 | combining | #1 AND #2 AND #3 |
| 5 | Limits- 2017 to present, no literature reviews or case reports | #4 AND [2017-2023]/py NOT ([editorial]/lim OR [review]/lim OR 'case report'/de) |

**Web of Science Search**

| Set # | Concept | Syntax |
| --- | --- | --- |
| 1 | Artificial Intelligence, machine learning, neural networks | TS=("Artificial Intelligence" OR "Neural Network" OR "Neural Networks" OR "Deep Learning" OR "Machine Learning" OR “algorithm*” OR “augmented intelligence”) |
| 2 | Dermatology and skin diseases | TS=(derm* OR skin OR psoriasis OR alopecia OR acne OR cutaneous) |
| 3 | Disease severity | TS=(severity OR severities OR assessment OR “patient acuity” OR measurement) |
| 4 | combining | #1 AND #2 AND #3 |
| 5 | Limits- 2017 to present, no literature reviews or case reports | #4 and 2023 or 2022 or 2021 or 2020 or 2019 or 2018 or 2017 (Publication Years) and Review Article or Editorial Material (Exclude – Document Types) |

**IEEE Search**

| Set # | Concept | Syntax |
| --- | --- | --- |
| 1 | Artificial Intelligence, machine learning, neural networks | "Artificial Intelligence" OR "Neural Network" OR "Neural Networks" OR "Deep Learning" OR "Machine Learning" OR “algorithm*” OR “augmented intelligence” |
| 2 | Dermatology and skin diseases | derm* OR skin OR psoriasis OR alopecia OR acne OR cutaneous |
| 3 | Disease severity | severity OR severities OR assessment OR “patient acuity” OR measurement |
| 4 | combining | #1 AND #2 AND #3 |
| 5 | Limits- 2017 to present, no literature reviews or case reports | #4 limited to 2017-2023 |

**Appendix 2. Preferred Reporting Items for a Systematic Review and Meta-analysis of Diagnostic Test Accuracy Studies (PRISMA-DTA)**

| **Section/topic** | **#** | **PRISMA-DTA Checklist Item** | **Reported on page #** |
| --- | --- | --- | --- |
| **TITLE / ABSTRACT** | | |  |
| Title | 1 | Identify the report as a systematic review (+/- meta-analysis) of diagnostic test accuracy (DTA) studies. | 1 |
| Abstract | 2 | Abstract: See PRISMA-DTA for abstracts. | 2 |
| **INTRODUCTION** | | |  |
| Rationale | 3 | Describe the rationale for the review in the context of what is already known. | 3, 4 |
| Clinical role of index test | D1 | State the scientific and clinical background, including the intended use and clinical role of the index test, and if applicable, the rationale for minimally acceptable test accuracy (or minimum difference in accuracy for comparative design). | 4 |
| Objectives | 4 | Provide an explicit statement of question(s) being addressed in terms of participants, index test(s), and target condition(s). | 4 |
| **METHODS** | | |  |
| Protocol and registration | 5 | Indicate if a review protocol exists, if and where it can be accessed (e.g., Web address), and, if available, provide registration information including registration number. | 5 |
| Eligibility criteria | 6 | Specify study characteristics (participants, setting, index test(s), reference standard(s), target condition(s), and study design) and report characteristics (e.g., years considered, language, publication status) used as criteria for eligibility, giving rationale. | 5 |
| Information sources | 7 | Describe all information sources (e.g., databases with dates of coverage, contact with study authors to identify additional studies) in the search and date last searched. | 4-5 |
| Search | 8 | Present full search strategies for all electronic databases and other sources searched, including any limits used, such that they could be repeated. | 4-5, Appendix 1 |
| Study selection | 9 | State the process for selecting studies (i.e., screening, eligibility, included in systematic review, and, if applicable, included in the meta-analysis). | 4-5 |
| Data collection process | 10 | Describe method of data extraction from reports (e.g., piloted forms, independently, in duplicate) and any processes for obtaining and confirming data from investigators. | 5 |
| Definitions for data extraction | 11 | Provide definitions used in data extraction and classifications of target condition(s), index test(s), reference standard(s) and other characteristics (e.g. study design, clinical setting). | 5 |
| Risk of bias and applicability | 12 | Describe methods used for assessing risk of bias in individual studies and concerns regarding the applicability to the review question. | 6 |
| Diagnostic accuracy measures | 13 | State the principal diagnostic accuracy measure(s) reported (e.g. sensitivity, specificity) and state the unit of assessment (e.g. per-patient, per-lesion). | 5-6 |
| Synthesis of results | 14 | Describe methods of handling data, combining results of studies and describing variability between studies. This could include, but is not limited to: a) handling of multiple definitions of target condition. b) handling of multiple thresholds of test positivity, c) handling multiple index test readers, d) handling of indeterminate test results, e) grouping and comparing tests, f) handling of different reference standards | 5-6 |
| Meta-analysis | D2 | Report the statistical methods used for meta-analyses, if performed. | 7-8 |
| Additional analyses | 16 | Describe methods of additional analyses (e.g., sensitivity or subgroup analyses, meta-regression), if done, indicating which were pre-specified. | 8 |
| **RESULTS** |  |  |  |
| Study selection | 17 | Provide numbers of studies screened, assessed for eligibility, included in the review (and included in meta-analysis, if applicable) with reasons for exclusions at each stage, ideally with a flow diagram. | Figure 1,  page 6 |
| Study characteristics | 18 | For each included study provide citations and present key characteristics including: a) participant characteristics (presentation, prior testing), b) clinical setting, c) study design, d) target condition definition, e) index test, f) reference standard, g) sample size, h) funding sources | Table 1 |
| Risk of bias and applicability | 19 | Present evaluation of risk of bias and concerns regarding applicability for each study. | Appendix 4,  page 7 |
| Results of individual studies | 20 | For each analysis in each study (e.g. unique combination of index test, reference standard, and positivity threshold) report 2x2 data (TP, FP, FN, TN) with estimates of diagnostic accuracy and confidence intervals, ideally with a forest or receiver operator characteristic (ROC) plot. | Pages 6-7,  Figures 2-4 |
| Synthesis of results | 21 | Describe test accuracy, including variability; if meta-analysis was done, include results and confidence intervals. | Pages 6-7,  Figures 2-4, Appendices 5 and 7 |
| Additional analysis | 23 | Give results of additional analyses, if done (e.g., sensitivity or subgroup analyses, meta-regression; analysis of index test: failure rates, proportion of inconclusive results, adverse events). | Page 7,  Tables 2-3, Appendices 6 and 8 |
| **DISCUSSION** |  |  |  |
| Summary of evidence | 24 | Summarize the main findings including the strength of evidence. | 7-8 |
| Limitations | 25 | Discuss limitations from included studies (e.g. risk of bias and concerns regarding applicability) and from the review process (e.g. incomplete retrieval of identified research). | 9 |
| Conclusions | 26 | Provide a general interpretation of the results in the context of other evidence. Discuss implications for future research and clinical practice (e.g. the intended use and clinical role of the index test). | 9 |
| **FUNDING** |  |  |  |
| Funding | 27 | For the systematic review, describe the sources of funding and other support and the role of the funders. | 6 |
| Meta-analysis | D2 | Report the statistical methods used for meta-analyses, if performed. | 7-8 |
| Additional analyses | 16 | Describe methods of additional analyses (e.g., sensitivity or subgroup analyses, meta-regression), if done, indicating which were pre-specified. | 8 |

*Adapted From:*  McInnes MDF, Moher D, Thombs BD, McGrath TA, Bossuyt PM, The PRISMA-DTA Group (2018). Preferred Reporting Items for a Systematic Review and Meta-analysis of Diagnostic Test Accuracy Studies: The PRISMA-DTA Statement. JAMA. 2018 Jan 23;319(4):388-396. doi: 10.1001/jama.2017.19163.

**Appendix 3. Studies included for meta-analysis**

| **Study** | **Skin Disease** | **Scoring Metric** | **Outcome Measure** |
| --- | --- | --- | --- |
| Bang et al (2021)[1] | Atopic Dermatitis | Eczema Area and Severity Index (EASI) | Accuracy |
| Cho et al (2022)[2] | Atopic Dermatitis | Investigator Global Assessment (IGA) for Atopic dermatitis | Accuracy |
| Folle et al (2023)[3] | Nail Psoriasis | Modified Nail Psoriasis Severity Index (mNAPSI) | Accuracy |
| Huynh et al (2022)[4] | Acne Vulgaris | IGA of Acne | Accuracy |
| Lim et al (2020)[5] | Acne Vulgaris | IGA of Acne | Accuracy |
| Ni et al (2022)[6] | Radiation Dermatitis | Radiation Therapy Oncology Group (RTOG) Grading Criteria for Skin Toxicity | Accuracy |
| Raj et al (2023)[7] | Psoriasis Vulgaris | Psoriasis Area Severity Index (PASI) | Accuracy |
| Raj et al (2021)[8] | Psoriasis Vulgaris | PASI | Accuracy |
| Ranjan et al (2021)[9] | Radiation Dermatitis | Common Terminology Criteria for Adverse Events (CTCAE) Grading for Skin Toxicity | Accuracy |
| Schaap et al (2022)[10] | Psoriasis Vulgaris | PASI | Accuracy and correlation (ICC) |
| Wang et al #1 (2023)[11] | Acne Vulgaris | Combined the Global Acne Grading System (GAGS) and Hayashi Grading Criterion | Accuracy |
| Wang et al #2 (2023)[12] | Acne Vulgaris | New Acne Severity Quantification Criterion (Modified GAGS) | Accuracy |
| Amruthalingam et al (2023)[13] | Hand Ezcema | Hand Eczema Severity Index (HECSI) | Correlation (ICC) |
| Amruthalingam et al (2022)[14] | Pustular Psoriasis | Palmoplantar Pustulosis Psoriasis Area Severity Index (PPPASI) | Correlation (ICC) |
| Fink et al (2019)[15] | Psoriasis Vulgaris | PASI | Correlation (ICC) |
| Lee et al (2023)[16] | Psoriasis Vulgaris | PASI | Correlation (ICC) |
| Lin et al (2022)[17] | Psoriasis Vulgaris | PASI | Correlation (ICC) |
| Meienberger et al (2020)[18] | Psoriasis Vulgaris | PASI | Correlation (ICC) |
| Yang et al (2021)[19] | Acne Vulgaris | Chinese Guidelines for the Management of Acne Vulgaris | Correlation (Kendall’s W) |

**Appendix 4. Summary of characteristics of included studies**

| **Author** | **Target condition** | **Severity score** | **Outcome**  **measures** | **AI model architecture** | **AI model interpretability** | **Validation**  **approach** |
| --- | --- | --- | --- | --- | --- | --- |
| Amruthalingam et al (2023)[13] | Hand ezcema | Hand Eczema Severity Index (HECSI) | Precision, sensitivity, and correlation (ICC) | U-Net with  ResNet  backbone | Anatomical stratification  report | Incomplete reporting of the training or testing datasets |
| Amruthalingam et al (2022)[14] | Pustular psoriasis | Palmoplantar Pustulosis Psoriasis Area Severity Index (PPPASI) | Correlation  for count and surface percentage  (ICC and SC) | U-Net and  Res-Net | Saliency map | Validation using data taken from the original dataset, validation set independent |
| Attar et al (2023)[20] | Atopic dermatitis | Six Area, Six Sign Atopic Dermatiis Score (SASSAD), Three Item Severity Score (TISS), Eczema Area and Severity Index (EASI) | Precision, F1 score, Root Mean Square Error (RMSE) | U-Net | N/A | Incomplete reporting of the training or testing datasets |
| Bang et al (2021)[1] | Atopic dermatitis | EASI | Accuracy, specificity, and sensitivity | ResNet V1, ResNet V2, GoogLeNet, VGG-Net | Text-based (image interpreted as the probability of each severity level) | Validation using data collected from a separate source to the training set |
| Bernardis et al (2018)[21] | Alopecia Areata | Severity of Alopecia Tool (SALT) | Mean Square Error (MSE), average shape match, average covering match | Not specified | Texture extraxtion to produce SALT score | Incomplete reporting of the training or testing datasets |
| Cho et al (2022)[2] | Atopic dermatitis | Investigator Global Assessment (IGA) for Atopic dermatitis | Accuracy, F1 score, and Youden index | ResNetV2 | N/A | Validation using data collected from a separate source to the training set |
| Fink et al (2019)[15] | Psoriasis vulgaris | Psoriasis Area Severity Index (PASI) | Precision, Mean Absolute Difference (MAD), Mean Relative Difference (MRD), and correlation (ICC) | Not specified | N/A | Incomplete reporting of the training or testing datasets |
| Folle et al (2023)[3] | Nail psoriasis | Modified Nail Psoriasis Severity Index (mNAPSI) | Accuracy, Area Under the Curve (AUC), sensitivity, specificity, accuracy, F1 score, and correlation | transformer-based network (BEiT18, 86M parameters) | N/A | Validation using data taken from the original dataset, validation set independent |
| Gao et al  (2022)[22] | Androgenic alopecia | Basic and specific (BASP) classification | Accuracy | D-Net and R-Net (CNN) | N/A | Validation using data taken from the original dataset, validation set independent |
| George et al (2020)[23] | Psoriasis vulgaris | PASI | Accuracy, F1 score, and correlation (ICC) | ML classifiers trained for scale severity scoring (support vector machine, random forests, zeroR classifier) | Bag-of-visual words model for lesion feature extractions using superpixels as keypoints, local binary patterns, histogram, compact composite descriptors | Incomplete reporting of the training or testing datasets |
| George et al (2018)[24] | Psoriasis vulgaris | PASI (Erythema) | Accuracy, RMSE, and F1 score | AlexNet | Dictionary learning techniques | Validation using data taken from the original dataset, validation set independent |
| Guo et al (2022)[25] | Vitiligo | Physician Global Assessment (PGA) Tool for Vitiligo, Vitiligo Area Scoring Index (VASI) | Sensitivity and error rate, Jaccard Index (JI) | Yolo v3, PSPNet, UNet, and UNet++ | Saliency maps | Validation using data taken from the original dataset, validation set independent |
| Hsieh et al (2022)[26] | Nail  psoriasis | Nail Psoriasis Severity Index (NAPSI) | Accuracy, sensitivity, specificity, and precision | ResNet | Saliency maps | Validation using data taken from the original dataset, validation set independent |
| Huang et al (2023)[27] | Psoriasis vulgaris | PASI | Mean average error (MAE) | EfficientNet-B0 | No | Validation using data taken from the original dataset, validation set independent |
| Huynh et al (2022)[4] | Acne  vulgaris | IGA of Acne | Accuracy, AUC, and Mean Average Precision (mAP) | Faster R-CNN, ResNet50 | No | Validation using data taken from the original dataset, validation set independent |
| Lee et al (2023)[16] | Psoriasis vulgaris | PASI | MAE and correlation (ICC) | Mask R- CNN with a Swin Transformer small backbone | Saliency maps  and text based of score | Validation using data taken from the original dataset, validation set independent |
| Lee et al (2020)[28] | Alopecia Areata | SALT, Topography-based Alopecia Areata Severity Tool (TOAST) | JI | U-Net | Saliency maps and text based of score | Validation using data taken from the original dataset, validation set independent |
| Lim et al (2020)[5] | Acne  vulgaris | IGA of Acne | Accuracy and correlation (Pearson coefficient) | CNN, DenseNet, OR Inception v4, ResNet18 | Text-based output (IGA score) | Validation using data taken from the original dataset, validation set independent |
| Lin et al (2023)[29] | Acne  vulgaris | IGA of Acne | Accuracy, sensitivity, specificity, precision, and Youden Index | VGG16, VGG11 Teacher–student structure | Text-based justification of count and score | Validation using data taken from the original dataset, validation set independent |
| Lin et al (2022)[17] | Psoriasis vulgaris | PASI (Area) | Accuracy, sensitivity, speciﬁcity, JI, and Dice coefficient (DSC), Correlation (ICC) | U-Net | Saliency maps | Validation using data taken from the original dataset, validation set independent |
| Li et al (2020)[30] | Psoriasis vulgaris | PASI | MAE and Pair Accuracy (PA) | PSENet (CNN) | Attention mask | Validation using data taken from the original dataset, validation set independent |
| Liu et al  (2022)[31] | Acne  vulgaris | Hayashi Grading Criterion | Sensitivity, specificity. precision, Youden Index | Ensemble learning algorithms | N/A | Validation using data taken from the original dataset, validation set independent |
| Medela et al (2022)[32] | Atopic dermatitis | SCORing Atopic Dermatitis (SCORAD) | Accuracy and RMAE | Legit.Health- SCORADNet (EfficientNet-B0-based CNN) | N/A | Validation using data collected from a separate source to the training set |
| Meienberger et al (2020)[18] | Psoriasis vulgaris | PASI (Area) | MAD and correlation (ICC) | Net16 (CNN) | N/A | Validation using data taken from the original dataset, validation set independent |
| Melina et al (2018)[33] | Acne vulgaris | IGA of Acne | Accuracy and correlation (Pearson coefficient) | Multilayer perceptron network (CNN) | N/A | Validation using data taken from the original dataset, validation set independent |
| Montilla et al (2023)[34] | Hidradenitis suppurativa | International Hidradenitis Suppurativa Severity Score System (IHS4) | MAE, precision,  and recall | YOLOv5 | CADx system | Validation using data taken from the original dataset, validation set independent |
| Moon et al (2022)[35] | Psoriasis vulgaris | PASI | Accuracy, precision, F1 score, and recall | RegNetY 1.6 G | Text-based justification | Incomplete reporting of the training or testing datasets |
| Ni et al (2022)[6] | Radiation Dermatitis | Radiation Therapy Oncology Group (RTOG) Grading Criteria for Skin Toxicity | Accuracy, precision, F1 score, and recall | DenseNet- 121 | Segmentation map | Validation using data taken from the original dataset, validation set independent |
| Okamoto et al (2022)[36] | Psoriasis vulgaris | PASI | MAE | Inception V3 | Grad-CAM visualization | Validation using data taken from the original dataset, validation set independent |
| Paik et al (2023)[37] | Palmoplantar Psoriasis | PPPASI | Accuracy, MAE, and correlation (ICC) | Attention  U-Net | Include map of area included in images | Validation using data taken from the original dataset, validation set independent |
| Polesie et al (2022)[38] | Melanoma | Breslow depth | AUC | ResNet-50 | N/A | Incomplete reporting of the training or testing datasets |
| Raj et al (2023)[7] | Psoriasis vulgaris | PASI (Area) | Accuracy and MAE | MobileNet V2 and U-Net | Segmentation map | Validation using data taken from the original dataset, validation set independent |
| Raj et al (2021)[8] | Psoriasis vulgaris | PASI (Area) | Accuracy | Modified U-Net | Segmentation map | Validation using data taken from the original dataset, validation set independent |
| Ranjan et al (2021)[9] | Radiation dermatitis | Common Terminology Criteria for Adverse Events (CTCAE) Grading for Skin Toxicity | Accuracy, sensitivity, and specificity | eCNN | Signal intensity mapping | Validation using prospectively collected data |
| Schaap et al (2022)[10] | Psoriasis vulgaris | PASI | Accuracy, MAE, and correlation (ICC) | CNNCORAL, ResNet-18 | Text-based justification | Validation using data taken from the original dataset, validation set independent |
| Seité et al (2019)[39] | Acne  vulgaris | Global Acne Severity Scale (GEA) | Correlation (ICC) | deep learning provided by Perfect Mobile Corp | N/A | Validation using data taken from the original dataset, validation set independent |
| Tancharoen et al (2019)[40] | Psoriasis vulgaris | PASI | Accuracy | Not specified | Segmentation map | Incomplete reporting of the training or testing datasets |
| Toh et al (2018)[41] | Vitiligo | VASI | Correlation (Pearson coefficient) | Not specified | N/A | Incomplete reporting of the training or testing datasets |
| Wada et al (2021)[42] | Radiation dermatitis | CTCAE Grading for Skin Toxicity | Accuracy, sensitivity, specificity, precision, and F1 score | DCNN and VGG-16 | Grad-CAM visualization | Validation using data taken from the original dataset, validation set independent |
| Wang et al #1 (2023)[11] | Acne  vulgaris | Combined the Global Acne Grading System (GAGS) and Hayashi Grading Criterion | Accuracy, precision, F1 score, and recall | Acne-RegNet (student)= LRegNet (teacher) (classification) MobileNet V3 (segmentation) | N/A | Validation using data taken from the original dataset, validation set independent |
| Wang et al #2 (2023)[12] | Acne  vulgaris | New Acne Severity Quantification Criterion (Modified GAGS) | Accuracy, sensitivity, specificity, and precision | Localization-DL: SE-ResNet-50 (teacher) MobileNetV2 (student) Classification: L-HRNet | N/A | Validation using data taken from the original dataset, validation set independent |
| Wen et al (2022)[43] | Acne  vulgaris | Hayashi Grading Criterion | Precision, recall, and Intersection over Union (IoU) | faster_rcnn_resnet101 | Object-level interpretability through bounding boxes + Text | Validation using data taken from the original dataset, validation set independent |
| Wu et al (2019)[44] | Acne  vulgaris | Hayashi Grading Criterion | Accuracy, sensitivity, speciﬁcity, precision, Youden Index, MAE, and MSE | ResNet-50 | Object-level interpretability through bounding boxes + score Text | Validation using data taken from the original dataset, validation set independent |
| Yang et al (2021)[19] | Acne  vulgaris | Chinese Guidelines for the Management of Acne Vulgaris | F1 score and correlation (Kendall’s W and kappa coefficient) | Inception v3 | Text-based justification | Validation using data taken from the original dataset, validation set independent |
| Zhang et al (2022)[45] | Acne  vulgaris | Hayashi Grading Criterion | Accuracy | ResNet | Prediction vs. true results maapping | Incomplete reporting of the training or testing datasets |

**Appendix 5. Additional information on study characteristics**

| **Author** | **Country** | **Study design** | **Data source** | **Reference standard**  **(ground truth)** | **Skin tone data** | **Demographics data** | **Class distribution and balance** | **Use of full body image** |
| --- | --- | --- | --- | --- | --- | --- | --- | --- |
| Amruthalingam et al (2022) | Switzerland | Retrospective | Hospital | Expert annotation (Two board-certified dermatologists and a student independently labeled the images for pustules and brown spots for the 151 standardized images) | No | No | No | No |
| Amruthalingam et al (2023) | Switzerland | Retrospective | Hospital | Expert annotation (11 experienced dermatologists annotated eczema lesions, healthy skin and background) | Yes | No | No | No |
| Attar et al (2023) | United Kingdom | Retrospective | Clinical trial data set | Expert annotation  (clinical staff recorded corresponding severity of seven disease signs and four dermatologists delineated AD regions at pixel-level) | Yes | Yes | No | No |
| Bang et al (2021) | South Korea | Retrospective | Hospital | Expert annotation  (Three dermatologists) | Yes | No | Yes | No |
| Bernardis et al (2018) | USA | Retrospective | Hospital | Expert annotation | No | Yes | No | No |
| Cho et al (2022) | South Korea | Retrospective | Hospital | Expert annotation  (Five dermatologists) | No | Yes | Yes | No |
| Fink et al (2019) | Germany | Retrospective | Hospital | Expert annotation  (Four dermatologists) | No | Yes | No | Yes |
| Folle et al (2023) | Germany | Retrospective | Hospital | Expert annotation  (Three rheumatologists) | No | Yes | Yes | No |
| Gao et al  (2022) | China | Retrospective | Hospital | Expert annotation  (Three dermatologists) | No | No | No | No |
| George et al (2018) | Australia | Retrospective | Hospital | Expert annotation  (The generated lesion ground truth from the 95 images has been verified and validated by three dermatologists) | No | No | No | Yes |
| George et al (2020) | Australia | Retrospective | Hospital | Expert annotation  (Three dermatologists) | No | No | No | Yes |
| Guo et al (2022) | China | Retrospective | Hospital | Expert annotation (Dermatologist labels) | Yes | No | No | No |
| Hsieh et al (2022) | Taiwan | Retrospective | Hospital and public dataset | Expert annotation (Dermatologists) | No | No | No | No |
| Huang et al (2023) | China | Retrospective | Hospital | Expert annotation  (Average of three dermatologists PASI scores) | No | No | No | No |
| Huynh et al (2022) | Vietnam | Retrospective | Smartphone App "Skin Detective" (private) | Expert annotation  (Consensus dermatologist labels and scores, senior last) | No | No | Yes | No |
| Lee et al (2020) | South Korea | Retrospective | Hospital | Expert annotation  (Pixelwise annotations of one dermatologist) | No | No | No | No |
| Lee et al (2023) | South Korea | Retrospective | Hospital | Expert annotation (Consensus dermatologist labels and scores using palm method) | Yes | No | No | No |
| Lim et al (2020) | Singapore | Retrospective | Hospital | Expert annotation (Dermatology resident, clinical scoring) | No | No | Yes | No |
| Lin et al (2023) | China | Retrospective | Mixed (ACNE04 and PLSBRACNE01) | Expert annotation (Dermatologist labels, counts and scores) | No | No | Yes | No |
| Lin et al (2022) | Taiwan | Retrospective | Hospital | Expert annotation (Dermatologists' markings of the psoriasis lesion border) | No | No | No | No |
| Li et al (2020) | China | Retrospective | Private images of patients through dermatologists | Expert annotation (Annotation by annotated by 11 professional dermatologists (nine professors and two attending physicians) | No | No | Yes | No |
| Liu et al (2022) | China | Retrospective | Public dataset (ACNE04) | Expert annotation (Dermatologists) | No | No | No | No |
| Medela et al (2022) | Spain | Retrospective | Public datasets (Dermatology Atlas, Danderm, Interactive Dermatology Atlas, DermIS, DermNet NZ and Hellenic Dermatological Atlas) | Expert annotation  (Nine experts, three for each dataset, who treat patients with AD in their daily practice, to reduce variability by combining their results) | Yes | Yes | Yes | No |
| Meienberger et al (2020) | Switzerland | Retrospective | University Hospital of Zurich | Expert annotation | No | No | No | No |
| Melina et al (2018) | Italy | Retrospective | Department of Dermatology, University of Magna Graecia | Expert annotation  (Majority voting among three experienced dermatologists) | No | Yes | Yes | No |
| Montilla et al (2023) | Spain | Retrospective | Public datasets (DermQuest and DermnetNZ) | Expert annotation | No | No | Yes | No |
| Moon et al (2022) | South Korea | Retrospective | Hospital | Expert annotation (Dermatologists) | No | No | No | No |
| Ni et al (2022) | China | Retrospective | 2 private data sets (Queen Mary Hospital, Hong Kong SAR, China and The Affiliated Cancer Hospital of Zhengzhou University, Zhengzhou, China and 1 online public database | Expert annotation | No | Yes | Yes | No |
| Okamoto et al (2022) | Japan | Retrospective | 16 Hospitals  in Japan | Expert annotation | No | No | No | No |
| Paik et al (2023) | South Korea | Retrospective | Seoul National University Bundang Hospital | Expert annotation | No | Yes | Yes | No |
| Polesie et al (2022) | Austria and Sweden | Retrospective | Sahlgrenska University Hospital | Expert annotation (histology) | No | No | Yes | No |
| Raj et al (2023) | India | Retrospective | Psoriasis Clinic and Research Centre, Psoriatreat, India | Expert annotation | No | No | No | No |
| Raj et al (2021) | India | Retrospective | Psoriasis Clinic and Research Centre, Psoriatreat, India | Expert annotation | No | No | No | No |
| Ranjan et al (2021) | Austria | Prospective | Department of Therapeutic Radiology and Oncology, Comprehensive Cancer Center, Graz, Austria | Expert annotation | Yes | Yes | Yes | No |
| Schaap et al (2022) | Netherland | Retrospective | Child-CAPTURE registry.  The Radboud university medical center, Nijmegen, Netherlands | Expert annotation  (Real-life PASI scores: the treating physician prior to capturing the images) | Yes | Yes | Yes | No |
| Seité et al (2019) | France, South Africa, China, India | Retrospective | Hospital | Expert annotation (Dermatologists) | No | Yes | Yes | No |
| Tancharoen et al (2019) | Thailand | Retrospective | Hospital | Expert annotation | No | No | No | No |
| Toh et al (2018) | Singapore | Retrospective | Hospital | Expert annotation (VASI scores of dermatologists or VASI scores in clinical trials) | No | No | No | No |
| Yang et al (2021) | China | Retrospective | Hospital | Expert annotation | Yes | No | Yes | No |
| Wada et al (2021) | Japan | Retrospective | Hospital | Expert annotation | No | No | Yes | No |
| Wang et al #1 (2023) | China | Retrospective | Mixed (ACNE04 and Hospital) | Expert annotation  (Consensus annotations and scores of dermatologists) | Yes | Yes | Yes | No |
| Wang et al #2 (2023) | China | Retrospective | Hospital | Expert annotation | No | Yes | Yes | No |
| Wen et al (2022) | China | Retrospective | ACNE04 | Expert annotation | No | No | Yes | No |
| Wu et al (2019) | China | Retrospective | ACNE04 | Expert annotation | No | No | Yes | No |
| Zhang et al (2022) | Singapore, China | Retrospective | ACNE04 | Expert annotation | No | No | Yes | No |

**Appendix 6. Number of images, capturing modalities and photography types of included studies**

|  | **Number of total images** | **Training data (Number of images)** | **Validation data (Number of images)** | **Testing data (Number of images)** | **Capturing modalities** | **Full body or regional photography** |
| --- | --- | --- | --- | --- | --- | --- |
| Amruthalingam et al (2022) | 151 | 121 | No validation | 30 | N/A | Regional |
| Amruthalingam et al (2023) | 312 | 249 | No validation | 63 | N/A | Regional |
| Attar et al (2023) | 1345 | 807 | 269 | 269 | Digital camera | Regional |
| Bang et al (2021) | 8000 | 5600 | No validation | 2400 | N/A | Regional |
| Bernardis et al (2018) | 250 | N/A | N/A | N/A | N/A | Regional |
| Cho et al (2022) | 9192 | 6623 | No validation | 2559 | N/A | Regional |
| Fink et al (2019) | 1920 | N/A | N/A | 1056 | Digital camera | Full body |
| Folle et al (2023) | 1154 | 738 | 185 | 231 | Tablet or smartphone | Regional |
| George et al (2018) | 676 | 541 | No validation | 135 | N/A | Full body |
| George et al (2020) | 839 | N/A | N/A | 96 | N/A | Full body |
| Guo et al (2022) | 4147 | 3186 | 398 | 563 | Digital camera | Regional |
| Hsieh et al (2022) | 460 | 300 | 150 | 10 | Custom built camera | Regional |
| Huang et al (2023) | 14096 | N/A | N/A | N/A | Digital camera or smartphone | Regional |
| Huynh et al (2022) | 1572 | 1100 | No validation | 472 | Smartphone | Regional |
| Lee et al (2020) | 3116 | 1901 | 815 | 400 | N/A | Regional |
| Lee et al (2023) | 2028 | 1731 | No validation | 297 | N/A | Regional |
| Lim et al (2020) | 472 | 314 | 60 | 98 | N/A | Regional |
| Lin et al (2023) | 1657 | 1325 | No validation | 332 | N/A | Regional |
| Lin et al (2022) | 255 | 165 | 41 | 49 | DSLR | Regional |
| Li et al (2020) | 5205 | 4164 | No validation | 1041 | N/A | Regional |
| Medela et al (2022) | 1083 | 503 | 101 | 479 | N/A | Regional |
| Meienberger et al (2020) | 259 | 162 | 41 | 56 | Digital camera | Regional |
| Melina et al (2018) | 2395 | 1435 | 480 | 480 | N/A | Regional |
| Montilla et al (2023) | 221 | 184 | No validation | 37 | N/A | Regional |
| Moon et al (2022) | 792 | 634 | No validation | 158 | N/A | Regional |
| Ni et al (2022) | 1205 | 841 | 182 | 182 | Digital camera | Regional |
| Okamoto et al (2022) | 705 | 556 | 139 | 10 | Digital camera | Regional |
| Paik et al (2023) | 701 | 550 | 61 | 90 | N/A | Regional |
| Polesie et al (2022) | N/A | N/A | N/A | 523 | N/A | Regional |
| Raj et al (2023) | 500 | 320 | 80 | 100 | Digital camera | Regional |
| Raj et al (2021) | 350 | 280 | 70 | No testing | Digital camera | Regional |
| Ranjan et al (2021) | 2263 | 1697 | No validation | 566 | Smartphone | Regional |
| Schaap et al (2022) | 1731 | 1385 | No validation | 346 | N/A | Regional |
| Seité et al (2019) | 6131 | 4958 | No validation | 1173 | Smartphone | Regional |
| Tancharoen et al (2019) | N/A | N/A | N/A | 10 | N/A | Regional |
| Toh et al (2018) | N/A | N/A | N/A | N/A | N/A | Regional |
| Yang et al (2021) | 1957 | 1565 | 392 | 40 | Digital camera | Regional |
| Wada et al (2021) | 752 | 647 | No validation | 105 | N/A | Regional |
| Wang et al #1 (2023) | 2970 | 2376 | 297 | 297 | Smartphone | Regional |
| Wang et al #2 (2023) | 1803 | 1099 | 137 | 567 | Smartphone | Regional |
| Wen et al (2022) | 1222 | 855 | 367 | No testing | N/A | Regional |
| Wu et al (2019) | 1457 | 1165 | No validation | 292 | Digital camera | Regional |
| Zhang et al (2022) | 1457 | 1165 | No validation | 292 | N/A | Regional |

**Appendix 7. Summary of methodological quality assessment of all 45 studies using modified QUADAS-2**

%

**Risk of Bias**

**Applicability Concerns**

**Appendix 8. Deek’s Regression Asymmetry Funnel Plot for accuracy**


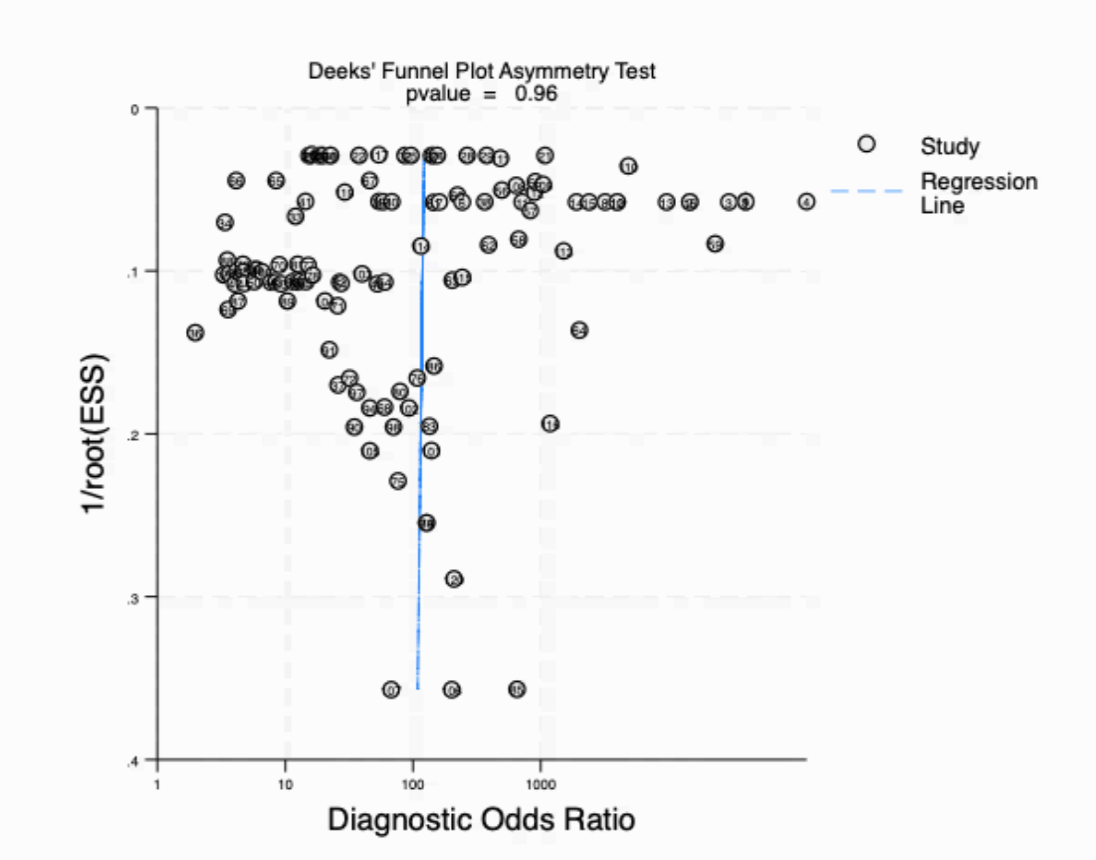


**Appendix 9. Sensitivity analyses for accuracy**

- When excluding low-quality studies

|  | **overall** | **excluding low Q** |
| --- | --- | --- |
| **Sensitivity (%)** | 80.5 (76.2-84.2) | 80.8 (74.4-85.9) |
| **Specificity (%)** | 96.2 (94.9-97.2) | 95.7 (93.5-97.2) |

- When excluding outliers

|  | **overall** | **excluding outliers** |
| --- | --- | --- |
| **Sensitivity (%)** | 80.5 (76.2-84.2) | 80.1 (76.6-83.2) |
| **Specificity (%)** | 96.2 (94.9-97.2) | 97.1 (96.4-97.6) |

- When excluding both low-quality studies and outliers

|  | **overall** | **excluding outliers and low-quality studies** |
| --- | --- | --- |
| **Sensitivity (%)** | 80.5 (76.2-84.2) | 85.4 (81.0-88.9) |
| **Specificity (%)** | 96.2 (94.9-97.2) | 95.8 (93.0-97.5) |

**Appendix 10. Results for reliability**

The pooled reliability was 78.0% (72.2-83.8) in disease severity assessment. Considerable heterogeneity (I^2^= 99.5%) suggested that the reliability varies by study. The asymmetrical funnel plot and Egger’s test results (p<0.0001) indicated that publication bias might be present. Consistently, the Duval and Tweedie method showed the possibility of publication bias, and a slightly increased overall reliability of 82.6% (76.4-88.8) was estimated if the bias was corrected. Excluding outliers resulted in a slightly decreased reliability of 75.2% (70.2-80.3), yet the heterogeneity meaningfully lowered (I^2^ = 76.5%). However, the pooled reliability significantly increased when the low-quality studies were excluded (89.1%, 82.8-95.4).

1. **Forest plot for reliability**


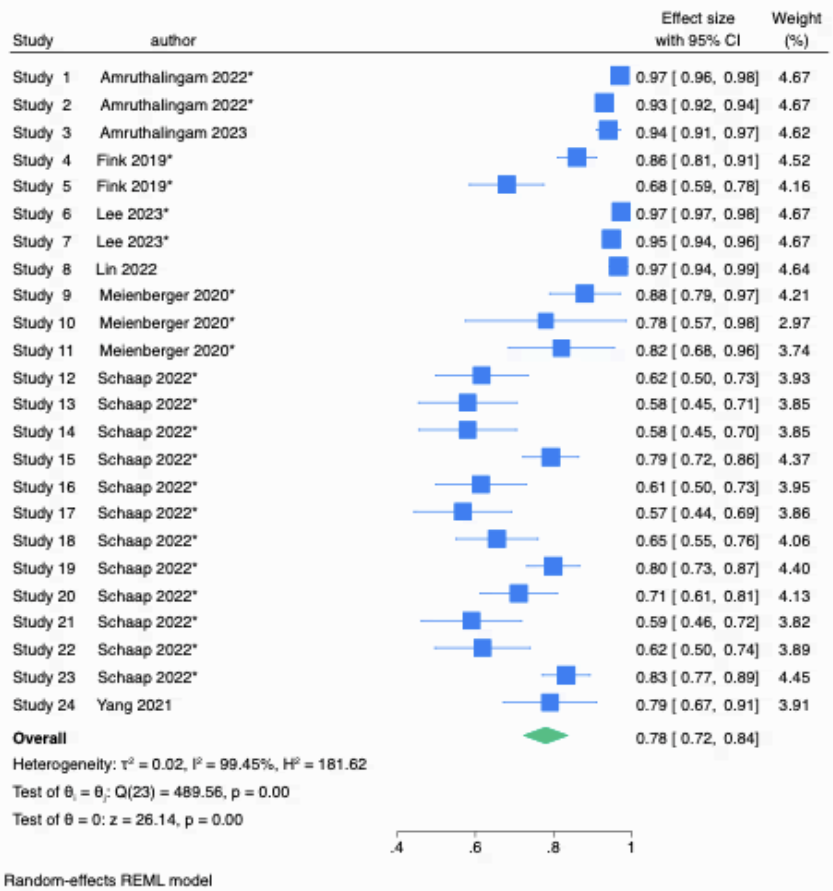


1. **Asymmetry funnel plot for reliability**


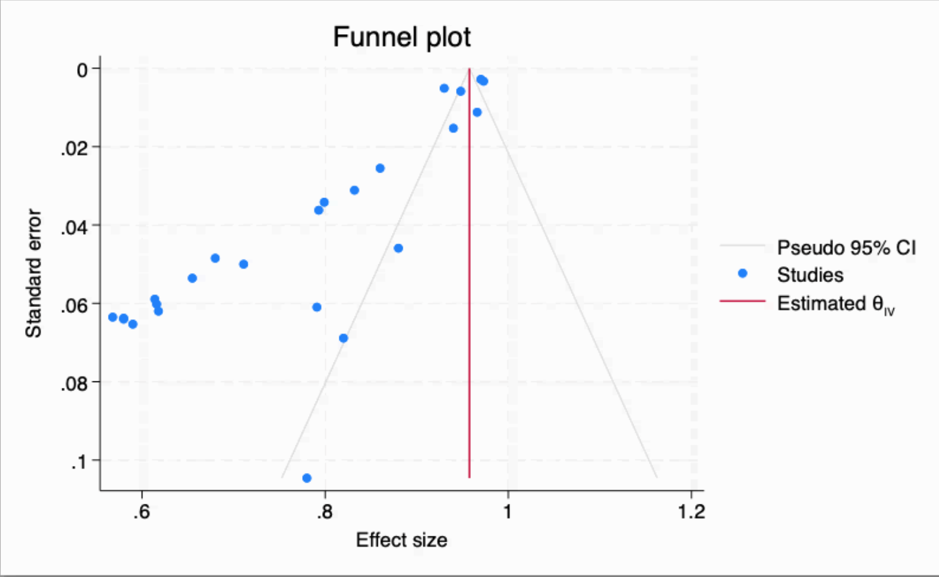


1. **Duval and Tweedie trim and fill method**

| Studies | Effect size (ICC) | 95% CI (ICC) |
| --- | --- | --- |
| Observed (n=24) | 0·78 | 0·72-0·84 |
| Observed+Imputed (n=29) | 0·83 | 0·76-0·89 |

*ICC: Intra-class correlation coefficient

1. **Sensitivity analyses for reliability**

|  | **Overall** | **Excluding low Q** | **Excluding outliers** |
| --- | --- | --- | --- |
| Reliability (ICC) | 0·78 (0·72-0·84) | 0·89 (0·83-0·95) | 0·75 (0·70-0·80) |

**Appendix 11. Studies excluded in the full-text review stage with reasons**

| **Author** | **Reason for exclusion** |
| --- | --- |
| Fink et al (2018) | Absence of AI |
| Flament et al (2019) | Photoaging |
| Gudobba et al (2022) | Non-validated severity score |
| Horikawa et al (2022) | Abstract only |
| Hurault et al (2022) | Does not assess disease severity |
| Lin et al (2022) | Duplicate |
| Lin et al (2022) | Duplicate |
| Nayagi et al (2023) | Does not assess disease severity |
| Park et al (2022) | Does not assess disease severity |
| Rahmad et al (2021) | Does not assess disease severity |
| Sacha et al (2021) | Does not assess disease severity |
| Thiboutot et al (2021) | Absence of AI |
| Toh et al (2018) | Non-validated severity score |
| Webster et al (2022) | Wrong study design |
| Lin et al (2021) | Duplicate |
| Xing et al (2023) | Not peer-reviewed |
| Khan et al (2017) | Unable to access |
| Marioni et al (2017) | Does not assess disease severity |

**References**

1. Bang CH, Yoon JW, Ryu JY, Chun JH, Han JH, Lee YB, Lee JY, Park YM, Lee SJ, Lee JH (2021) Automated severity scoring of atopic dermatitis patients by a deep neural network. Sci Rep 2021 111 11:1–8

2. Cho SI, Lee D, Han B, Lee JS, Hong JY, Chung JH, Lee DH, Na JI (2023) Practical Training Approaches for Discordant Atopic Dermatitis Severity Datasets: Merging Methods With Soft-Label and Train-Set Pruning. IEEE J Biomed Health Inform 27:166–175

3. Folle L, Fenzl P, Fagni F, et al (2023) DeepNAPSI multi-reader nail psoriasis prediction using deep learning. Sci Rep 2023 131 13:1–8

4. Huynh QT, Nguyen PH, Le HX, et al (2022) Automatic Acne Object Detection and Acne Severity Grading Using Smartphone Images and Artificial Intelligence. Diagn 2022 Vol 12 Page 1879 12:1879

5. Lim ZV, Akram F, Ngo CP, Winarto AA, Lee WQ, Liang K, Oon HH, Thng STG, Lee HK (2020) Automated grading of acne vulgaris by deep learning with convolutional neural networks. Skin Res Technol 26:187–192

6. Ni R, Zhou T, Ren G, Zhang Y, Yang D, Tam VCW, Leung WS, Ge H, Lee SWY, Cai J (2022) Deep Learning-Based Automatic Assessment of Radiation Dermatitis in Patients With Nasopharyngeal Carcinoma. Int J Radiat Oncol Biol Phys 113:685–694

7. Raj R, Londhe ND, Sonawane R (2023) PsLSNetV2: End to end deep learning system for measurement of area score of psoriasis regions in color images. Biomed Signal Process Control 79:104138

8. Raj R, Londhe ND, Sonawane RS (2021) Deep Learning based Multi-Segmentation for Automatic Estimation of Psoriasis Area Score. Proc 8th Int Conf Signal Process Integr Netw SPIN 2021 1137–1142

9. Ranjan R, Partl R, Erhart R, Kurup N, Schnidar H (2021) The mathematics of erythema: Development of machine learning models for artificial intelligence assisted measurement and severity scoring of radiation induced dermatitis. Comput Biol Med 139:104952

10. Schaap MJ, Cardozo NJ, Patel A, de Jong EMGJ, van Ginneken B, Seyger MMB (2022) Image-based automated Psoriasis Area Severity Index scoring by Convolutional Neural Networks. J Eur Acad Dermatol Venereol 36:68–75

11. Wang J, Luo Y, Wang Z, Hounye AH, Cao C, Hou M, Zhang J (2023) A cell phone app for facial acne severity assessment. Appl Intell 53:7614–7633

12. Wang J, Wang C, Wang Z, Hounye AH, Li Z, Kong ML, Hou M, Zhang J, Qi M (2023) A novel automatic acne detection and severity quantification scheme using deep learning. Biomed Signal Process Control 84:104803

13. Amruthalingam L, Mang N, Gottfrois P, Gonzalez Jimenez A, Maul JT, Kunz M, Pouly M, Navarini AA (2023) Objective hand eczema severity assessment with automated lesion anatomical stratification. Exp Dermatol 32:521–528

14. Amruthalingam L, Buerzle O, Gottfrois P, Jimenez AG, Roth A, Koller T, Pouly M, Navarini AA (2022) Quantification of Efflorescences in Pustular Psoriasis Using Deep Learning. Healthc Inform Res 28:222–230

15. Fink C, Alt C, Uhlmann L, Klose C, Enk A, Haenssle HA (2019) Precision and reproducibility of automated computer‐guided Psoriasis Area and Severity Index measurements in comparison with trained physicians. Br J Dermatol 180:390–396

16. Lee WH, Lee S, Kim J, Han JH, Kim YH, Kim J, Lee JH, Bang CH (2023) Measurement of psoriasis-affected area with artificial neural network. J Am Acad Dermatol 88:731–732

17. Lin YL, Huang A, Yang CY, Chang WY (2022) Measurement of Body Surface Area for Psoriasis Using U-net Models. Comput Math Methods Med. https://doi.org/10.1155/2022/7960151

18. Meienberger N, Anzengruber F, Amruthalingam L, Christen R, Koller T, Maul JT, Pouly M, Djamei V, Navarini AA (2020) Observer-independent assessment of psoriasis-affected area using machine learning. J Eur Acad Dermatol Venereol 34:1362–1368

19. Yang Y, Guo L, Wu Q, et al (2021) Construction and Evaluation of a Deep Learning Model for Assessing Acne Vulgaris Using Clinical Images. Dermatol Ther 11:1239–1248

20. Attar R, Hurault G, Wang Z, Mokhtari R, Pan K, Olabi B, Earp E, Steele L, Williams HC, Tanaka RJ (2023) Reliable Detection of Eczema Areas for Fully Automated Assessment of Eczema Severity from Digital Camera Images. JID Innov 3:100213

21. Bernardis E, Castelo-Soccio L (2018) Quantifying Alopecia Areata via Texture Analysis to Automate the SALT Score Computation. J Investig Dermatol Symp Proc 19:S34–S40

22. Gao M, Wang Y, Xu H, Xu C, Yang X, Nie J, Zhang Z, Li Z, Hou W, Jiang Y (2022) Deep Learning-based Trichoscopic Image Analysis and Quantitative Model for Predicting Basic and Specific Classification in Male Androgenetic Alopecia. Acta Derm Venereol. https://doi.org/10.2340/ACTADV.V101.564

23. George Y, Aldeen M, Garnavi R (2020) Automatic Scale Severity Assessment Method in Psoriasis Skin Images Using Local Descriptors. IEEE J Biomed Health Inform 24:577–585

24. George Y, Aldeen M, Garnavi R (2018) Psoriasis image representation using patch-based dictionary learning for erythema severity scoring. Comput Med Imaging Graph 66:44–55

25. Guo L, Yang Y, Ding H, Zheng H, Yang H, Xie J, Li Y, Lin T, Ge Y (2022) A deep learning-based hybrid artificial intelligence model for the detection and severity assessment of vitiligo lesions. Ann Transl Med 10:590–590

26. Hsieh KY, Chen HY, Kim SC, Tsai YJ, Chiu HY, Chen GY (2022) A mask R-CNN based automatic assessment system for nail psoriasis severity. Comput Biol Med 143:105300

27. Huang K, Wu X, Li Y, et al (2023) Artificial Intelligence-Based Psoriasis Severity Assessment: Real-world Study and Application. J Med Internet Res. https://doi.org/10.2196/44932

28. Lee S, Lee JW, Choe SJ, Yang S, Koh SB, Ahn YS, Lee WS (2020) Clinically Applicable Deep Learning Framework for Measurement of the Extent of Hair Loss in Patients With Alopecia Areata. JAMA Dermatol 156:1018–1020

29. Lin Y, Jiang J, Chen D, Ma Z, Guan Y, Liu X, You H, Yang J (2023) DED: Diagnostic Evidence Distillation for acne severity grading on face images. Expert Syst Appl 228:120312

30. Li Y, Wu Z, Zhao S, et al (2020) PSENet: Psoriasis Severity Evaluation Network. Proc AAAI Conf Artif Intell 34:800–807

31. Liu S, Fan Y, Duan M, Wang Y, Su G, Ren Y, Huang L, Zhou F (2022) AcneGrader: An ensemble pruning of the deep learning base models to grade acne. Skin Res Technol 28:677–688

32. Medela A, Mac Carthy T, Aguilar Robles SA, Chiesa-Estomba CM, Grimalt R (2022) Automatic SCOring of Atopic Dermatitis Using Deep Learning: A Pilot Study. JID Innov 2:100107

33. Artificial Intelligence for the Objective Evaluation of Acne Investigator Global Assessment - JDDonline - Journal of Drugs in Dermatology. https://jddonline.com/articles/artificial-intelligence-for-the-objective-evaluation-of-acne-investigator-global-assessment-S1545961618P1006X. Accessed 17 Feb 2024

34. Hernández Montilla I, Medela A, Mac Carthy T, et al (2023) Automatic International Hidradenitis Suppurativa Severity Score System (AIHS4): A novel tool to assess the severity of hidradenitis suppurativa using artificial intelligence. Skin Res Technol 29:e13357

35. Moon CI, Lee J, Kye S, Baek YS, Lee O (2022) Federated Learning for Masked Psoriasis Severity Classification. Proc IEEE Sens. https://doi.org/10.1109/SENSORS52175.2022.9967333

36. Okamoto T, Kawai M, Ogawa Y, Shimada S, Kawamura T (2022) Artificial intelligence for the automated single-shot assessment of psoriasis severity. J Eur Acad Dermatol Venereol 36:2512–2515

37. Paik K, Kim BR, Youn SW (2023) Evaluation of the area subscore of the Palmoplantar Pustulosis Area and Severity Index using an attention U-net deep learning algorithm. J Dermatol 50:787–792

38. Polesie S, Gillstedt M, Kittler H, Rinner C, Tschandl P, Paoli J (2022) Assessment of melanoma thickness based on dermoscopy images: an open, web-based, international, diagnostic study. J Eur Acad Dermatol Venereol 36:2002–2007

39. Seité S, Khammari A, Benzaquen M, Moyal D, Dréno B (2019) Development and accuracy of an artificial intelligence algorithm for acne grading from smartphone photographs. Exp Dermatol 28:1252–1257

40. Tancharoen D, Tantawiwat P, Kovintavewat P (2019) Medical Imaging using Automatic Region of Interest Segmentation for Psoriasis Diagnosis. 34th Int Tech Conf CircuitsSystems Comput Commun ITC-CSCC 2019. https://doi.org/10.1109/ITC-CSCC.2019.8793310

41. Toh JJH, Bhoi S, Tan VWD, Chuah SY, Jhingan A, Kong AWK, Thng STG (2018) Automated scoring of vitiligo using superpixel‐generated computerized digital image analysis of clinical photographs: a novel and consistent way to score vitiligo. Br J Dermatol 179:220–221

42. Wada K, Watanabe M, Shinchi M, Noguchi K, Mukoyoshi T, Matsuyama M, Arimura T, Ogino T (2021) ハイブリッド生成法によるディープラーニングを用いた放射線皮膚炎グレード判定システムに関する研究. 日本放射線技術学会雑誌 77:787–794

43. Wen H, Yu W, Wu Y, Zhao J, Liu X, Kuang Z, Fan R (2022) Acne detection and severity evaluation with interpretable convolutional neural network models. Technol Health Care 30:143–153

44. Wu X, Wen N, Liang J, Lai YK, She D, Cheng MM, Yang J (2019) Joint acne image grading and counting via label distribution learning. Proc IEEE Int Conf Comput Vis 2019-October:10641–10650

45. Zhang H, Ma T (2022) Acne Detection by Ensemble Neural Networks. Sens 2022 Vol 22 Page 6828 22:6828
